# Marine Peroxy Sesquiterpenoids Induce Apoptosis by Modulation of Nrf2-ARE Signaling in HCT116 Colon Cancer Cells

**DOI:** 10.3390/md16100347

**Published:** 2018-09-23

**Authors:** Junsei Taira, Haruna Miyazato, Katsuhiro Ueda

**Affiliations:** 1 Department Bioresource Technology, Okinawa National College of Technology, 905 Henoko, Nago-city Okinawa Prefecture 905-2192, Japan; miyazato@gmail.com; 2 Department of Chemistry, Biology and Marine Science, University of the Ryukyus, 1 Senbaru, Nishihara-cho, Okinawa 903-2013, Japan; kueda@sci.u-ryukyu.ac.jp

**Keywords:** free radical, peroxy sesquiterpenoid, apoptosis, Nrf2-ARE signaling, HCT116 colon cancer cells, soft coral, *Sinularia* sp.

## Abstract

Our current study demonstrated that the marine peroxy sesquiterpenoids isolated from the Okinawan soft coral *Sinularia* sp. have an antitumor activity in human colon cancer cell (HCT) 116 colon cancer cells with their induction of apoptosis due to H_2_O_2_ production derived from the compounds. This study clarified that peroxy sesquiterpenoids (**1** and **2**) inhibited anti-apoptosis proteins, such as B-cell lymphoma-extra large (Bcl-xL) and phosphoAkt (pAkt). In addition, the heme oxygenase-1 (HO-1), nuclear factor-erythroid-2-related factor (Nrf2), and phosphoNrf2 (pNrf2) proteins related to the cell survival regulation signal of Nrf2-ARE (antioxidant response element) were also suppressed in the presence of these compounds. While the cells treated with the compounds and trolox as an antioxidant expressed the inhibited proteins, such as HO-1, Nrf2, and Bcl-xL, it was suggested that the H_2_O_2_ involving free radical reactions derived from the molecule would be a trigger of apoptosis with the modulation of Nrf2-ARE signaling in the cells.

## 1. Introduction

The soft coral genus *Sinularia* is known to have a number of bioactive sesquiterpenoids [1] similar to that of marine fungus [2]. Particularly, the cadinane-type sesquiterpenoids have various bioactivities, such as a cytotoxicity [3,4] and an anti-inflammatory effect [5,6]. Our current study demonstrated that the marine peroxy sesquiterpenoids isolated from the Okinawan soft coral *Sinularia* sp. collected from Irabu Island, Okinawa, Japan have an antitumor activity in HCT116 colon cancer cells [7,8]. 

Nrf2-ARE (Nrf2, nuclear factor-erythroid-2-related factor; ARE, antioxidant response element) signaling is a cell survival response to avoid cell damage against oxidative stress involving the excess production of reactive oxygen species (ROS) and reactive nitrogen species (RNS) or electrophiles. The dissociation of the Nrf2 from the Kelch-like ECH-associated protein 1 (Keap1) by oxidative stress and electrophiles as a consequence of the Keap1 cysteine thiol modification induces the DNA sequences located in the promoter and enhancer regions as ARE-mediated phase II detoxifying/antioxidant enzymes, such as NAD(P)H:-quinone oxidoreductase-1, glutathione *S*-transferase, thioredoxin, and heme oxygenase-1 (HO-1) [9]. Recently, the constitutive stabilization of Nrf2 was found in various human cancers that accelerate the expression of the detoxifying/antioxidant enzymes and lead to the proliferation of cells [9,10]. Thus, studying the Nrf2 inhibitor could be a way of fighting cancers. This study describes how the marine peroxy sesquiterpenoids induce apoptosis due to the suppression of Nrf2-ARE signaling in HCT116 colon cancer cells. 

## 2. Results

### 2.1. Peroxy Sesquiterpenoids 

**Figure 1:**
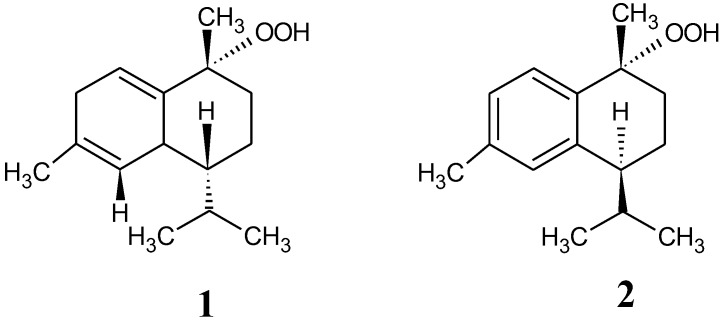
Chemical structures of marine peroxy sesquiterpenoids (**1** and **2**). The compounds were isolated from the Okinawan soft coral *Sinularia* sp.

**Figure 2:**
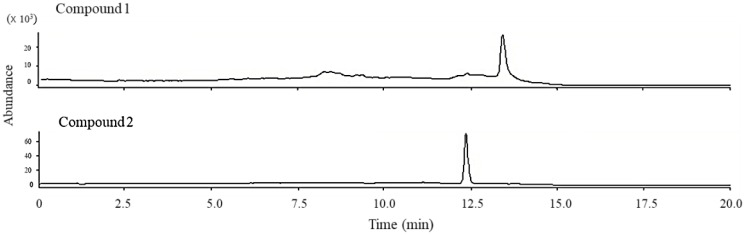
Total ion chromatogram obtained from peroxy sesquiterpenoids (**1** and **2**).

Peroxy sesquiterpenoids (**1**, **2**) in Figure 1 were isolated from the Okinawan soft coral *Sinularia* sp., as described in a previous study [7]. The total ion chromatogram of these compounds (**1**, **2**) was determined by LC/MS spectrometry in the selective ion mode (Figure 2). Each molecular weight (M + H)^+^ of compounds **1** (237.1) and **2** (235.1) was determined by LC/MS spectrometry in a selective positive ion mode. 

### 2.2. Nrf2 Protein Expression

**Figure 3:**
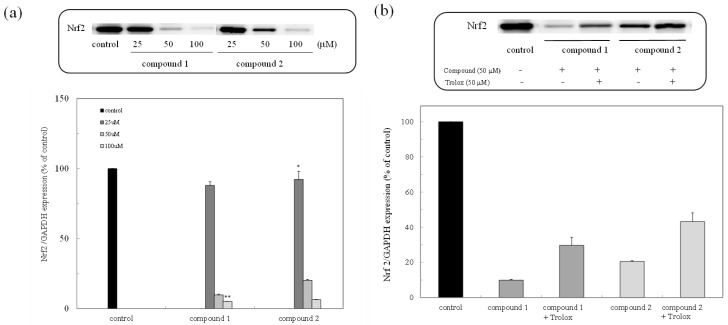
Expression of the nuclear factor-erythroid-2-related factor (Nrf2) protein due to peroxy sesquiterpenoids in HCT116 colon cancer cells. (**a**) Western blot analysis of the Nrf2 protein in the presence of compounds **1** and **2** (above) and densitometry analysis of the expression of the Nrf2 protein (below). Data were expressed as means ± SD. A Student’s *t*-test was applied to analyze the significance of the difference. * *p* < 0.05 and ** *p* < 0.01 indicated a significant difference from the control. (**b**) Western blot analysis of the Nrf2 protein in the presence of trolox (above) and densitometry analysis of the expression of the Nrf2 protein (below).

The expression of the Nrf2 protein in the colon cancer cells was examined through Western blot analysis. As shown in Figure 3a, the expression of the Nrf2 protein was inhibited in a dose-dependent manner, while the inhibited Nrf2 protein was expressed due to the administration of trolox as an antioxidant (Figure 3b).

### 2.3. HO-1 Protein Expression 

**Figure 4:**
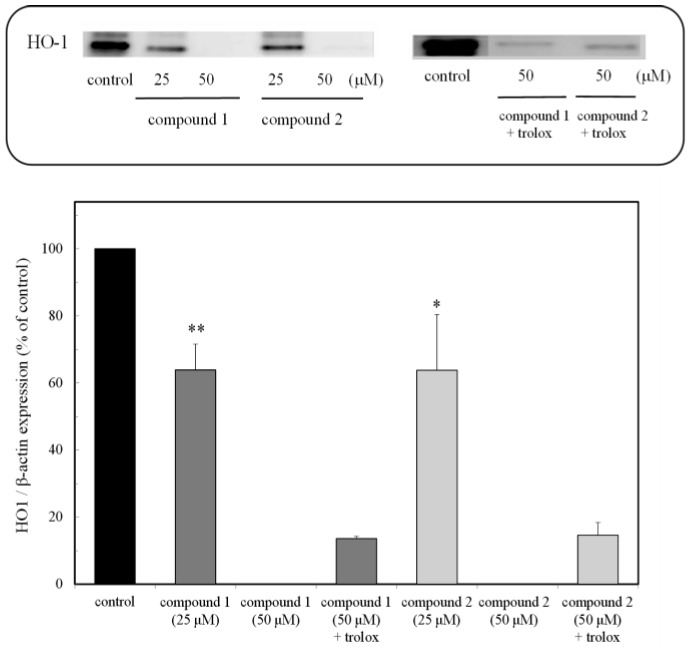
Expression of the HO-1 protein due to peroxy sesquiterpenoids in HCT116 colon cancer cells. Western blot analysis of the HO-1 protein in the presence of the compounds, with and without trolox (above). Densitometry analysis of the expression of the HO-1 protein (below). Data were expressed as means ± SD. A Student’s *t*-test was applied to analyze the significance of the difference. * *p* < 0.05 and ** *p* < 0.01 indicated a significant difference from the control.

The expression of the HO-1 protein through the Nrf2-ARE pathway was examined with and without compounds. HO-1 expression in cells was suppressed in the presence of the compounds, and the high concentration of treated cells (50 μM) completely inhibited the expression of the HO-1 protein (Figure 4). The cells treated with trolox as well as the compounds were expressed in the inhibited HO-1 protein. The result was similar to that of the Nrf2 expression, supporting the fact that the compounds inhibited the Nrf2-ARE pathway.

### 2.4. Bcl-xL Protein Expression 

**Figure 5:**
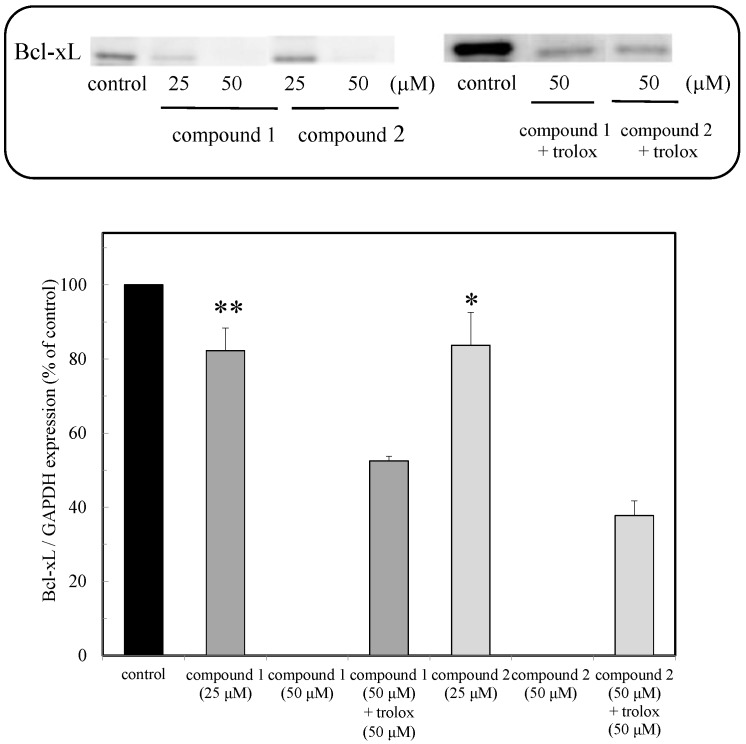
Expression of the Bcl-xL protein due to peroxy sesquiterpenoids in HCT116 cancer cells. Western blot analysis of the Bcl-xL protein in the presence of the compounds, with and without trolox (above). Densitometry analysis of the expression of the Bcl-xL protein (below). Data were expressed as means ± SD. A Student’s *t*-test was applied to analyze the significance of the difference. * *p* < 0.05 and ** *p* < 0.01 indicated a significant difference from the control.

The Bcl-xL protein is part of the Bcl-2 family of proteins that are known as anti-apoptosis proteins and are involved in the inhibition of caspase activation. In this study, the expression of the Bcl-xL protein was examined in the presence of compounds **1** and **2**. As shown in Figure 5, the compounds inhibited the expression of Bcl-xL, similarly to how the expression of the Nrf2 and HO-1 proteins was inhibited. The results indicated that the suppression of the expression of the Bcl-xL protein in the presence of the compounds leads to the induction of apoptosis through caspase activation (Figure 5).

### 2.5. pNrf2 and pAkt Expression

**Figure 6:**
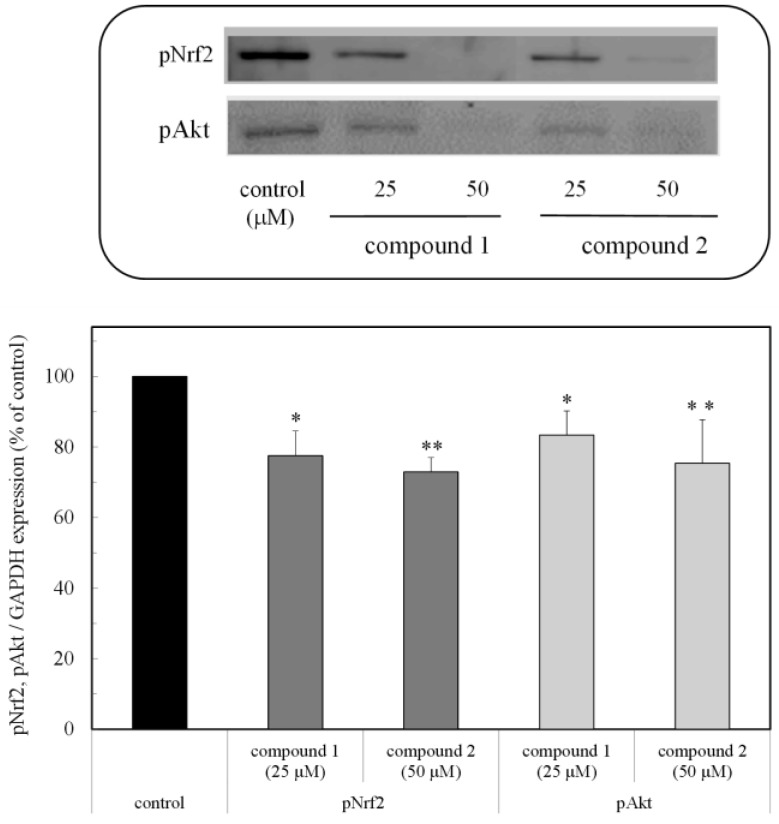
Expression of the pNrf2 and pAkt proteins due to peroxy sesquiterpenoids in HCT116 colon cancer cells. Western blot analysis of the pNrf2 and pAkt proteins in the presence of the compounds (above). Densitometry analysis of the expression of the pNrf2 and pAkt proteins (below). Data were expressed as means ± SD. A Student’s *t*-test was applied to analyze the significance of the difference. * *p* < 0.05 and ** *p* < 0.01 indicated a significant difference from the control.

As shown in Figure 6, the expression of the pNrf2 and pAkt proteins was inhibited in the presence of the compounds. This result suggested that the peroxy sesquiterpenoids inhibited Nrf2 activation related to the PI3K-Akt pathway, resulting in the induction of apoptosis due to the suppression of the Bcl-xL protein through caspase activation [10] (Figure 5).

## 3. Discussion

The peroxy sesquiterpenoids isolated from the Okinawan soft coral *Sinulalia* sp. exhibited several bioactivities, such as a cytotoxicity in human colon cancer cells and an anti-inflammatory action in macrophage cells (Figure 1) [7]. In this continuous study, we found that these compounds induced an antitumor activity (IC50 (μM) values as 50% cell death concentrations: 61.22 (compound **1**) and 43.73 (compound **2**)) by inducing apoptosis in HCT116 colon cancer cells due to the production of H_2_O_2_ by the compounds through a free radical reaction [8]. The constitutive stabilization of Nrf2 in colon cancer cells plays a significant role in the cytoprotection against cytotoxic compounds such as cancer drugs. The expression of the Nrf2 protein in the colon cancer cells was examined in the presence of the compounds (**1** and **2**). The expression of the Nrf2 protein was then inhibited while the inhibited Nrf2 protein was expressed due to the administration of trolox as an antioxidant (Figure 3). The expression of the HO-1 protein through the Nrf2-ARE pathway was also suppressed in the presence of the compounds (**1** and **2**). Furthermore, the cells treated with both the compounds and trolox were expressed in the inhibited HO-1 protein. The result was similar to that of the Nrf2 expression, supporting the fact that the compounds inhibited the Nrf2-ARE pathway. Our previous study showed that the intracellular H_2_O_2_ accumulated in cells in the presence of the peroxy sesquiterpenoids contributes to the induction of apoptosis through a free radical reaction [8]. As indicated in the letters, the production of H_2_O_2_ derived from the peroxy sesquiterpenoids plays a significant role as a trigger of caspase activation, with ensuing apoptosis. Thus, the consequence of H_2_O_2_ production involving free radical reactions derived from the compounds (**1** and **2**) may contribute to the elimination of Nrf2 stabilization. The apoptosis-inducing mechanisms may be relevant for the suppression of Nrf2-ARE-signaling.

The Bcl-2 family of proteins is characterized by its involvement in the regulation of apoptotic cell death. It consists of anti-apoptotic and pro-apoptotic members. The Bcl-xL protein is one member of the Bcl-2 family that are known as anti-apoptosis proteins involved in the inhibition of caspase activation. The intracellular H_2_O_2_ caused a disruption in the mitochondrial membrane potential, which led to the release of cytochrome c and then induced apoptosis. In this study, the expression of the Bcl-xL protein was examined in the presence of compounds **1** and **2** (Figure 5). Suppression of the expression of the Bcl-xL protein in the presence of the compounds led to apoptosis induction through caspase activation. Suppression of the expression of the Bcl-xL protein due to the administration of trolox supported the fact that the production of H_2_O_2_ involving a free radical contributed to the induction of apoptosis. 

Based on these results as well as previous studies, the H_2_O_2_ derived from the peroxy sesquiterpenoids may play an important role as a trigger of apoptosis through Nrf2 modulation. Nrf2 is known to play another role in cancer cells. It directly or indirectly facilitates the metabolic pathways and cell proliferation through PI3K-Akt signaling [11,12]. The Akt protein also regulates apoptosis through the inhibition of apoptosis related to proteins, such as Bcl-2, Bad, and Bcl-xL [13]. In this study, the expression of active types of phosphorous proteins, such as pNrf2 and pAkt, was examined in the presence of the compounds. The expression of these proteins was inhibited in the presence of the peroxy sesquiterpenoids (Figure 6). In our previous study, marine carotenoids, such as fucoxanthin and fucoxanthinol, induced apoptosis, then the expressions of pAkt, including Bcl-xL, were suppressed as anti-apoptotic proteins [14]. The compounds inhibited the expression of pAkt, which may induce apoptosis due to the downregulation of the PI3K-Akt signaling involved in Nrf2 phosphorylation. Presumably, the peroxy sesquiterpenoids may also have a similar function of apoptosis induction through the suppression of the anti-apoptotic proteins.

Thus, the results of this study suggest that the peroxy sesquiterpenoids inhibited the Nrf2 activation related to the PI3K-Akt pathway, resulting in the induction of apoptosis due to the suppression of the Bcl-xL protein through caspase activation. In addition, H_2_O_2_ will be a key molecule to study in order to elucidate the specificity of the compounds’ toxic effects, which may function as a second messaging signal or directly reduce proteins.

## 4. Materials and Methods

### 4.1. Materials

The products of anti-Nrf2 (Santa Cruz Biotechnology, Inc., Dallas, TX, USA), anti-HO-1 (StressMarq Biosciences Inc., Cadboro Bay, VIC, Canada), anti-Bcl-xL (BioVision, Inc., Milpitas, CA, USA), anti-phospho Akt (Cell Signaling Technology Japan, Inc., Tokyo, Japan), and anti-β-actin (Wako Pure Chemical Industries, Ltd., Osaka, Japan) were used for detecting protein expression. 

### 4.2. Cell Culture

HCT116 colon cancer cells (American Type Culture Collection, Manassas, VA, USA) were cultured in Dulbecco’s Modified Eagle Medium (DMEM) (including 10% fetal bovine serum (FBS), 100 U/mL penicillin, and 100 μg/mL streptomycin) at 37 °C in a 5% CO_2_ atmosphere. 

### 4.3. Western Blot Analysis

Compounds dissolved in DMSO were assayed by Western blot analysis. The cells were washed with phosphate buffered saline (PBS) and then treated with the lysis buffer. The cellular lysates were centrifuged at 13,800× *g* for 5 min. The total cellular extracts were separated on SDS-polyacrylamide gels (4–12% SDS-polyacrylamide; Thermo Fisher Scientific K.K, Yokohama, Japan) and transferred to a nitrocellulose membrane (iBlot Gel Transfer Mini; Thermo Fisher Scientific K.K, Yokohama, Japan ) using an iBlot Gel Transfer Device (Thermo Fisher Scientific K.K, Yokohama, Japan). Protein detection was carried out using an immunodetection system (Invitrogen, Thermo Fisher Scientific K.K, Yokohama, Japan) with the antibodies. The protein concentration of the cells was determined using a BCA protein assay kit (Thermo Fisher Scientific K.K, Yokohama, Japan).

### 4.4. LC/MS Analysis

Each sample was measured by LC/MS (Agilent1200, Agilent Technologies) using a photodiode array detector and was monitored at 280 nm on a reversed-phase chromatographic column (YMC-Pack Pro C18, 100 × 4.6 mm I.D., 5 μm particle size, YMC Co., Ltd., Kyoto, Japan) at 40.0 °C. The mobile phase consisting of a 5 mM formic acid aqueous solution (20%) and acetonitrile was carried out at the flow rate of 0.8 mL/min by a linear gradient to 50% (8 min) and 100% (5 min) and held for 5 min. The mass spectra were measured under the following conditions: electrospray ionization positive ion mode; desolvation temperature, 350 °C; desolvation pressure, 35 psig; desolvation gas flow, 12.01 mL/min (6120 Quadrupole, Agilent Technologies).

### 4.5. Statistical Analysis

The data were expressed as means ± SD. Significance: *p* < 0.05 and *p* < 0.01 were considered statistically significant. 

## 5. Conclusions

This study elucidated that the peroxy sesquiterpenoids induce apoptosis in HCT116 colon cancer cells due to the suppression of anti-apoptosis proteins and the cytoprotective activity of Nrf2-ARE signaling with the expression of the HO-1 protein. The reduction of the proteins was protected by the administration of trolox, indicating that the H_2_O_2_ production involving free radical reactions derived from the molecule could play an important role as a trigger of apoptosis through Nrf2 modulation.

## References

[1] Chen W., Li Y., Guo Y. (2012). Terpenoids of *Sinularia* soft corals: Chemistry and bioactivity. Acta Pharm. Sin. B.

[2] Zhu H., Hua X.X., Gong T., Pang J., Hou Q., Zhu P. (2013). Hypocreaterpenes A and B, cadinane-type sesquiterpenes from a marine-derived fungus, *Hypocreales* sp.. Phytochem. Lett..

[3] Yang B., Liao S., Lin X., Wang J., Liu J., Zhou X., Yang X., Liu Y. (2013). New sinularianin sesquiterpenes from soft coral *Sinularia* sp.. Mar. Drugs.

[4] Su J.H., Huang C.Y., Li P.J., Lu Y., Wen Z.H., Kao Y.H., Sheu J.H. (2012). Bioactive cadinane-type compounds from the soft coral *Sinularia scabra*. Arch. Pharm. Res..

[5] Li Y.C., Xian Y.F., Ip S.P., Su Z.R., Su J.Y., He J.J., Xie Q.F., Lai X.P., Lin Z.X. (2011). Anti-inflammatory activity of patchouli alcohol isolated from Pogostemonis Herba in animal models. Fitoterapia.

[6] Taira J., Tsuchida E., Uehara M., Kinjyo Y., Roy P.K., Ueda K. (2015). Dual biological functions of the apoptotic activity and anti-inflammatory effect by alcyonolide congeners from the Okinawan soft coral, *Cespitularia* sp.. Bioorg. Med. Chem. Lett..

[7] Roy P.K., Ashimine R., Miyazato H., Taira J., Ueda K. (2016). Endoperoxy and hydroperoxy cadinane-type sesquiterpenoids from an Okinawan soft coral, *Sinularia* sp.. Arch. Pharmacal. Res..

[8] Miyazato H., Taira L., Ueda K. (2016). Hydrogen peroxide derived from marine peroxy sesquiterpenoids induces apoptosis in HCT116 human colon cancer cells. Bioorg. Med. Chem. Lett..

[9] Kaspar J.W., Niture S.K., Jaiswal A.K. (2009). Nrf2:INrf2 (Keap1) signaling in oxidative stress. Free Radic. Biol. Med..

[10] Mitsuishi Y., Taguchi K., Kawatani Y., Shibata T., Nukiwa T., Aburatani H., Yamamoto M., Motohashi H. (2012). Nrf2 redirects glucose and glutamine into anabolic pathways in metabolic Reprogramming. Cancer Cell.

[11] DeBerardinis R.J., Lum J.J., Hatzivassiliou G., Thompson C.B. (2008). Nrf2 redirects glucose and glutamine into anabolic pathways in metabolic reprogramming. Cell Metab..

[12] Chandra J., Samali A., Orrenius S. (2000). Triggering and modulation of apoptosis by oxidative stress. Free Radic. Biol. Med..

[13] Franke T.F., Hornik C.P., Segev L., Shostak G.A., Sugimoto C. (2003). PI3K/Akt and apoptosis: Size matters. Oncogene.

[14] Taira J., Sonamoto M., Uehara M. (2017). Dual biological functions of a cytoprotective effect and apoptosis induction by boavailable marine carotenoid fucoxanthinol through modulation of the Nrf2 activation in RAW264.7 macrophage cells. Mar. Drugs.

